# Antiepileptic Effects of Cicadae Periostracum on Mice and Its Antiapoptotic Effects in H_2_O_2_-Stimulated PC12 Cells via Regulation of PI3K/Akt/Nrf2 Signaling Pathways

**DOI:** 10.1155/2021/5598818

**Published:** 2021-07-20

**Authors:** Qing Zhang, Ruo-Lan Li, Ting Tao, Jia-Yi Sun, Jia Liu, Ting Zhang, Wei Peng, Chun-Jie Wu

**Affiliations:** ^1^School of Pharmacy, Chengdu University of Traditional Chinese Medicine, Chengdu 611130, China; ^2^Innovation Research Institute, Chengdu University of Traditional Chinese Medicine, Chengdu 610075, China

## Abstract

Cicadae Periostracum (CPM), a commonly used animal traditional Chinese medicine (TCM), possesses antifebrile, spasmolytic, antiasthmatic, and antiphlogistic effects. In our present paper, we aimed to systemically investigate the antiepileptic effects of CPM in epileptic mice and explore the related molecular mechanism. Pentylenetetrazole- (PTZ) and strychnine-induced convulsion mice were established, and the results showed CPM could prolong the latency of convulsion and death and improve the neuronal damage in the hippocampus of PTZ-induced mice. Furthermore, the H_2_O_2_-treated PC12 cells were prepared to explore the possible mechanisms for the antiepileptic effects of CPM. CCK-8 results showed that CPM significantly improved the cell viability of H_2_O_2_-treated PC12 cells. Results of the acridine orange- (AO-) ethidium bromide (EB) staining, cell mitochondrial membrane potential (MOMP) analysis, and flow cytometry analysis showed that CPM significantly suppressed the H_2_O_2_-induced apoptosis in PC12 cells. In addition, CPM also downregulated the proapoptosis proteins, including Bax, cleaved- (C-) caspase-3, and C-caspase-9, and upregulated Bcl-2. Furthermore, CPM reduced the reactive oxygen species (ROS) levels via increasing antioxidative enzyme activities, including superoxide dismutase (SOD), catalase (CAT), and glutathione peroxidase (GSH-Px). Importantly, CPM could increase the phosphorylation of phosphoinositide 3-kinase (PI3K) and protein kinase B (Akt) in H_2_O_2_-induced PC12 cells and can promote the nuclear transfer of the nuclear factor E2-related factor 2 (Nrf2) and increase the expression of heme oxygenase-1 (HO-1) in the cytoplasm. In conclusion, our present study suggested CPM possessed antiepileptic effects through antiapoptosis of neuron cells via regulation of the PI3K/Akt/Nrf2 signaling pathway.

## 1. Introduction

Epilepsy is one of the predominant serious chronic neurological disorders in the clinic, and it is estimated that there are over 70,000,000 people subjected to epilepsy worldwide [1, 2]. The characteristics of epilepsy is spontaneous seizures; epilepsy is the result of multiple and complex causes and could lead to convulsions and cognitive dysfunction [1, 3]. It is reported that approximately 80% of the epileptic patients live in low- or middle-income areas, and the treatment of epilepsy requires long time and costly living care [1, 4]. Consequently, although great improvements have been achieved in the diagnosis and treatment of epilepsy, epilepsy remains a poorly curative disease in the clinic. Furthermore, almost all antiepileptic drugs available have safety and efficacy deficits, and thus, epileptic patients are usually reluctant to administrate these antiepileptic drugs [5]. Consequently, it is necessary to discover more reliable antiepileptic agents with less side effects for the clinical treatment of epilepsy.

**Figure 1:**
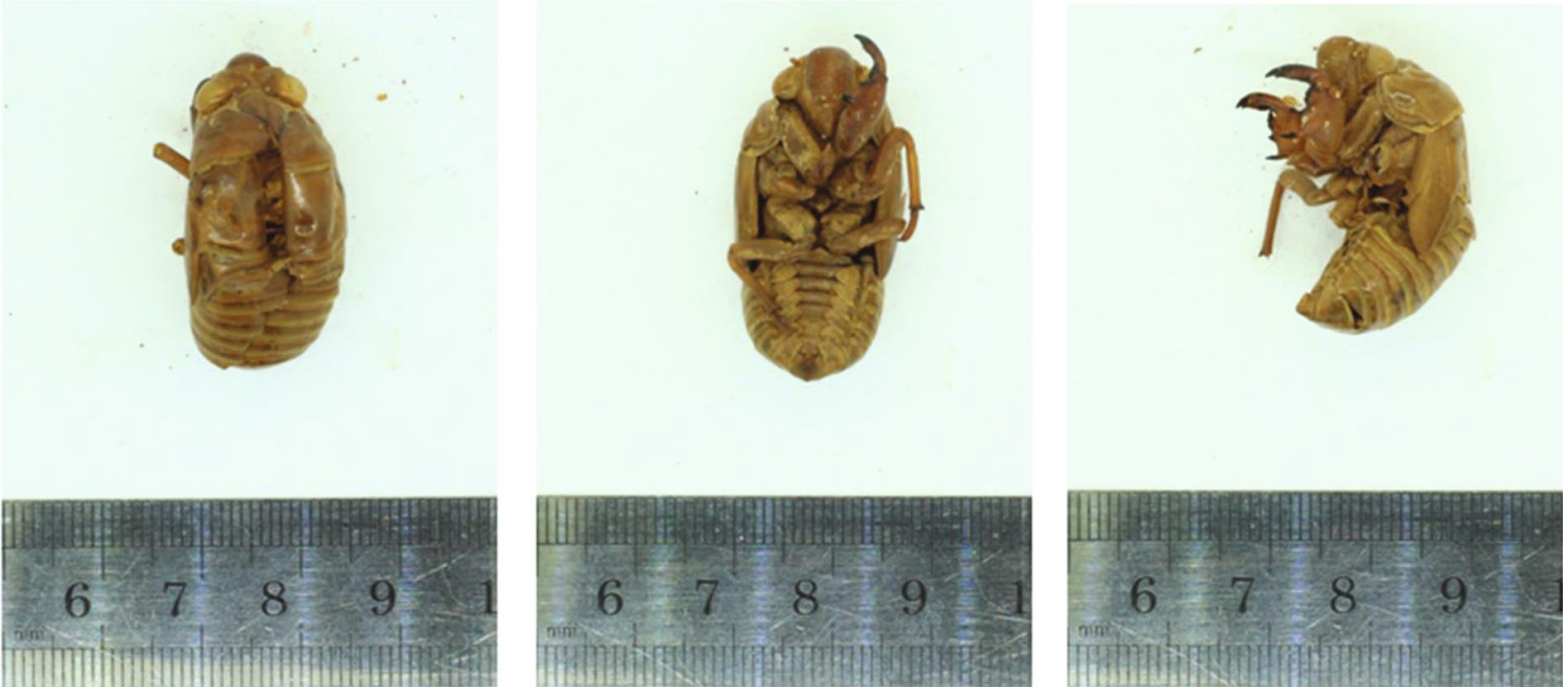
Cicadae Periostracum.

Cicadae Periostracum (CPM, Figure 1), the cast-off shell of the *Cryptotympana pustulata*, is a known animal traditional Chinese medicine (TCM) with the functions of “*dispelling wind and heat*,” “*removing nebula and improving vision*,” and “*expelling wind and relieving convulsion*”, etc. [6, 7]. Increasing investigations have revealed that acetyldopamine polymers and phenols are the predominant phytochemical constituents in CPM [8]. In addition, modern pharmacological evidences suggest that this animal TCM possesses a wide spectrum of pharmacological activities, including antifebrile, spasmolytic, antiasthmatic, and antiphlogistic effects [8, 9]. In the year of 2008, an experimental paper studied the effects of CPM on pentylenetetrazole- (PTZ-) induced mice and found that the water extracts of CPM had an antiepileptic activity [10]. However, the related possible molecular mechanism studies of the antiepileptic effects of CPM are lacking, which seriously limit the drug development of CPM. As part of our continuing work on animal TCMs with antiepileptic effects [11, 12], we found that CPM had significant antiepileptic effect in mice in our preliminary experiment. In our present paper, we aimed to systemically study the antiepileptic effects of CPM in experimental epileptic mouse models and further explore the potential molecular mechanism, which would be beneficial for the antiepileptic drug development of CPM in the future.

## 2. Materials and Methods

### 2.1. Materials and Chemicals

Dulbecco's modified Eagle's medium (DMEM) and antibiotics (penicillin and streptomycin) were purchased from the Hyclone Co. (Shanghai, China); phosphate-buffered saline (PBS) and trypsin-EDTA were purchased from GIBCO (Grand Island, NY, USA); the TransSerum® EQ Fetal Bovine Serum (FBS) was purchased from TransGen Biotech (Beijing, China); prestained protein marker was obtained from the Accurate Biotechnology (Hunan) Co., Ltd. (Hunan, China); RAPI buffer, nuclear protein extraction kit, BCA protein assay reagent, Bcl-2, and *β*-actin were purchased from the Boster Biological Technology Company (Wuhan, China); acridine orange- (AO-) ethidium bromide (EB) staining kit was obtained from Beyotime (Haimen, China); Annexin V-FITC/PI kit was purchased from Multisciences (Lianke) Biotechnology Corporate Limited (Hangzhou, China); polyvinylidene fluoride (PVDF) membrane and H_2_O_2_ were purchased from Sigma-Aldrich (Shanghai, China); CCK-8 kits, 4′,6-diamidino-2-phenylindole (DAPI), phalloidin, JC-1 probe, and DCFH-DA fluorescent probe were obtained from US Everbright® Inc. (Suzhou, China); enhanced chemiluminescence (ECL) luminescence reagent and cell cycle detection kit were purchased from Beijing 4A Biotech Co. (Beijing, China); assay kits for superoxide dismutase (SOD) and malondialdehyde (MDA) were purchased from the Nanjing Jiancheng Bioengineering Institute (Nanjing, China); assay kits for catalase (CAT) and glutathione peroxidase (GSH-Px) were obtained from Suzhou Michy Biomedical Technology Co., Ltd. (Suzhou, China); primary antibodies for cleaved caspase-3, cleaved caspase-9, and Bax were purchased from the Cell Signaling Technology Co. (Danvers, MA, USA); and protein kinase B (Akt), phosphorylation- (p-) Akt, and phosphoinositide 3-kinase (PI3K), p-PI3K, heme oxygenase-1 (HO-1), nuclear factor E2-related factor 2 (Nrf2), and goat-anti-rabbit/rat horseradish-peroxidase-conjugated (HRP) secondary antibodies were purchased from Abcam (Cambridge, MA, USA).

### 2.2. Preparation of CPM Freeze-Dried Powder

Cicadae Periostracum (CPM) was purchased from the Kangmei Pharmaceutical Co. (Puning, China) and identified by Professor Chun-Jie Wu from the School of Pharmacy of Chengdu University of Traditional Chinese Medicine (Chengdu, China). The medicinal material specimens were stored in the Herbal Medicine Specimen Hall of the School of Pharmacy of Chengdu University of Traditional Chinese Medicine (CP2020-0405). The CPM was extracted with water three times to obtain the water extract. The extract is filtered and evaporated using a rotating vacuum evaporator (EYELA, N-1300V, Tokyo, Japan), and then, the filtrates were freeze-dried using a lyophilizer (Labconco Co., Kansas, MI, USA). The obtained freeze-dried powder is sealed at 4°C and stored for future experimental use.

### 2.3. HPLC-Q Exactive Orbitrap-MS Assays

The main chemical constituents in the water extract of CPM were determined by HPLC-Q Exactive Orbitrap-MS. Chromatographic separation was carried out on an HPLC-Q Exactive Orbitrap-MS system (Thermo Fisher, Waltham, MA, USA) with a CAPCELL PAK C_18_ column (250 mm × 4.6 mm, i.d. 5 *μ*m, Shiseido, Japan). The separation was performed at 25°C using a gradient elution, and acetonitrile (A) and 0.1% formic acid-water (B) were used as the mobile phase. The gradient program was set as 0-10 min, 2%-13% A; 10-28 min, 13%-37% A; and 28-50 min, 37% A. The flow rate and injection volume were set as 0.2 mL/min and 10 *μ*L, respectively. For the mass analysis, nitrogen was applied as auxiliary gas. The mass determination was carried out based on positive and negative scanning modes with the m/z ranging from 100 to 1000.

### 2.4. Animals

**Figure 2:**
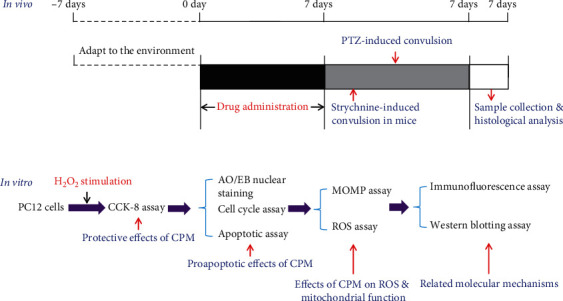
Experimental procedure of the *in vivo* and *in vitro* studies in this paper.

Male KM mice (18-22 g) were purchased from Chengdu Dasuo Experimental Animal Co., Ltd. (Chengdu, China). Before the formal experiment, the animals were fed adaptively for 7 days under 20 ± 2°C and 12 h light/12 h dark cycle environment and could take food and water freely. All the experimental protocols (Figure 2) in this study were strictly implemented in accordance with the International Laboratory Animal Care and Use Code and approved by the Animal Care and Use Committee of Chengdu University of Traditional Chinese Medicine (Chengdu, China).

### 2.5. Pentylenetetrazole- (PTZ-) Induced Convulsion in Mice

The anticonvulsant effect of the CPM was evaluated by pentylenetetrazole- (PTZ-) induced convulsion in mice as described in a previous paper [13]. A total of 100 healthy KM mice were randomly divided into 5 groups (*n* = 20), including the model group (mice were treated with normal saline, 20 mL/kg, p.o.), positive group (mice were treated with diazepam, 2.5 mg/kg/day, p.o., once), and three tested CPM groups (mice were treated with CPM at the doses of 100, 200, and 400 mg/kg/day, p.o.). Mice were administered with these drugs orally for one week, and sixty minutes after the final drug administration, mice in all groups were treated with PTZ (90 mg/kg) subcutaneously (s.c.). Then, the latency of the start of first convulsion and death was recorded during 30 minutes, and the convulsion rate and mortality were calculated.

### 2.6. Strychnine-Induced Convulsion in Mice

The anticonvulsant effect of the CPM was evaluated by strychnine-induced convulsion in mice as described in a previous paper [13]. A total of 120 healthy KM mice were randomly divided into 6 groups (*n* = 20), including the normal group (mice were treated with normal saline, 20 mL/kg, p.o.), model group (mice were treated with normal saline, 20 mL/kg, p.o.), positive group (mice were treated with diazepam, 2.5 mg/kg/day, p.o., once), and three tested CPM groups (mice were treated with CPM at the doses of 100, 200, and 400 mg/kg/day, p.o.). Mice were administered with these drugs orally for one week, and sixty minutes after the final drug administration, mice were injected intraperitoneally with strychnine (2.0 mg/kg) except for the normal group. Then, the latency of the start of first convulsion and death was recorded during 30 minutes, and the convulsion rate and mortality were calculated.

### 2.7. Histological Analysis

After being induced by PTZ, the seizure mice were anaesthetized with pentobarbital sodium (45 mg/kg, i.p.) and subsequently sacrificed by decapitation. Then, the whole brain was collected and fixed in 4% paraformaldehyde for 48 h. After ethanol dehydration, paraffin embedding, and slicing in 5 *μ*m, H&E staining and Nissl staining were carried out according to the methods described in the previous paper [14]. The pathological changes were observed using a Nikon TS2 optical microscope and high-resolution digital camera system (Nikon TS2, Tokyo, Japan).

### 2.8. Cell Culture

The rat pheochromocytoma-derived cell line PC12 cells were purchased from Wuhan Pu-nuo-sai Life Technology Co. Ltd. (Wuhan, China) and cultured in DMEM supplemented with 10% FBS at 37°C in a humidified 5% CO_2_ atmosphere. The cells were replaced with new media every two days, the cells were subcultured every three days, and the 3-5 generations of cells were used for the experiment in this study.

### 2.9. Cell Viability Test

The CCK-8 assay (Beyotime, Haimen, China) was used to detect cell viability to determine the optimal concentration and time of H_2_O_2_-treated PC12 cell death, as well as the optimal intervention concentration of CPM. In brief, PC12 cells in the logarithmic growth phase were inoculated into 96-well plates (1 × 10^4^ cells/well). After the cells were completely attached to the wall, different concentrations of PCM (20–600 *μ*g/mL) were added for further culture for 24 h, or different concentrations of H_2_O_2_ were added for 1-4 h. After intervention, a new cell medium was replaced, then the 10 *μ*L CCK-8 solution was added into each well and cultured at 37°C for another 1 h in the dark. Finally, the absorbance of each well was detected by using a multidetection iMARK microplate reader (Bio-Rad, Hercules, CA, USA). Under the 450 nm wavelength, and the cell survival rate was calculated. When determining the effects of CPM on H_2_O_2_-induced PC12 cells, we first pretreated the PC12 cells with CPM for 24 h, then treated the cells with a determined dose and time of H_2_O_2_, and finally detected the viability of cells after intervention.

### 2.10. AO/EB Staining

Acridine orange (AO) can cross intact cell membranes, embed into nuclear DNA, and emit bright green fluorescence at the 488 nm wavelength. In addition, ethidium bromide (EB) could only cross the damaged cell membrane, embed nuclear DNA, and exhibits orange fluorescence at the 515 nm wavelength [15]. Therefore, AO/EB double staining could be used to detect the effect of CPM therapy on the morphology of H_2_O_2_-induced PC12 cells. In brief, PC12 cells (1 × 10^5^) were inoculated in a confocal laser dish. After the cells were completely attached to the wall, the cells were treated with different concentrations of CPM for 24 h, followed by induction with H_2_O_2_. After induction, 20 *μ*L AO/EB staining solution was added for 2 min in the dark, and the excess staining solution was washed by PBS to remove. Finally, these images were observed and recorded by a laser confocal microscope (Leica, SP8 SR, Wetzlar, Germany).

### 2.11. Apoptosis Assay by Flow Cytometer

The apoptotic PC12 cells treated by H_2_O_2_ were determined by flow cytometry analysis using an Annexin V-FITC/PI kit. The cells in the logarithmic growth phase (1 × 10^5^) were seeded into a six-well plate and pretreated with different concentrations of CPM for 24 h and followed by incubation with H_2_O_2_. At the end of the experiment, the cells were collected and washed twice with precooled PBS; then, the supernatant was discarded by centrifugation. The cells were resuspended with 500 *μ*L binding buffer and then incubated with 5 *μ*L Annexin V-FITC and 5 *μ*L PI at room temperature for 15 min. Finally, the apoptosis rate was measured by flow cytometry (BD, New York, NY, USA).

### 2.12. Detection of Cell Cycle

Cells were seeded into a 6-well plate and then were treated with different concentrations of CPM and H_2_O_2_. After treatment, the cells were collected and washed with precooled PBS for 3 times. Then, the cells were resuspended with precooled 75% ethanol and fixed overnight at -20°C. Ethanol was discarded and the cells were washed, and then, 50 g/mL PI was added and stained for 30 min. Cell cycle distribution was analyzed by flow cytometry (BD, New York, NY, USA).

### 2.13. Mitochondrial Membrane Potential (MOMP) Determination

The cells were inoculated in laser confocal dishes (1 × 10^4^/well), treated with different concentrations of CPM for 24 h, and subsequently induced with H_2_O_2_. After the intervention, the 500 *μ*L JC-1 probe (10 *μ*g/mL) was added and incubated in the dark for 20 minutes. A confocal laser microscopy was used to observe the green and the red fluorescence (Leica, SP8 SR, Wetzlar, Germany) of cells at 530 nm and 590 nm, respectively, and the images were recorded.

### 2.14. Determination of Reactive Oxygen Species (ROS)

The DCFH-DA fluorescent probe was used to detect the level of intracellular reactive oxygen species (ROS). In brief, PC12 cells were inoculated in a confocal laser dish (1 × 10^4^/well), treated with different concentrations of CPM and H_2_O_2_, and incubated at 37°C for 30 min with proper amount of the DCFH-DA (10 *μ*M) probe. Fluorescence in cells was observed by laser confocal microscopy, and images were recorded (Leica, SP8 SR, Wetzlar, Germany).

### 2.15. Determination of Activities of Antioxidative Enzyme

The PC12 cells were seeded and cultured in 6-well plates for 12 h, then treated with different concentrations of CPM and H_2_O_2_. After the intervention, the supernatant was discarded, and the cells were washed with precooled PBS for 3 times. Then, RAPI was used to lyse the cells, and the total protein of the cells was collected. The concentration of the total protein of the cells was determined using the BCA detection kit. Finally, the activities of SOD, CAT, and GSH-Px and the levels of MDA in the cells were determined according to the standard protocols of the commercial kits.

### 2.16. Immunofluorescence Assay of Nrf2 Nuclear Translocation

The PC12 cells were seeded into a confocal laser dish and treated as previously described [16]. At the end of the intervention, the cells were washed with precooled PBS for 3 times and then fixed with paraformaldehyde without methanol for 20 min. Then, the cells were washed with PBS for 3 times and subsequently incubated with the blocking solution containing 0.3% Triton X-100 at room temperature for 1 hour. The primary antibody of Nrf2 (dilution of 1 : 300) was added and incubated overnight at 4°C. After washing with PBS for 3 times, anti-rabbit IgG (H+L) Alexa Fluor® 448 was added and incubated at room temperature for 1 h in the dark. The unbound secondary antibody was washed out by PBS, and an appropriate amount of phalloidin was added and incubated for 20 min at room temperature in the dark. Finally, the nuclei were stained with DAPI. Then, the Nrf2 nuclear translocation and fluorescence intensity were observed using a confocal laser microscope (Leica, SP8 SR, Wetzlar, Germany).

### 2.17. Western Blot Assay

As previously described, PC12 cells were seeded into a six-well plate, followed by intervention with PC and H_2_O_2_. After the intervention, a RAPI buffer and a nuclear protein extraction kit were used to extract total cell proteins, cellular proteins, and nuclear proteins, and the protein concentrations were detected. Similarly, after PTZ induction, the hippocampal tissues of mice were homogenized to extract the total protein and detect the protein concentration. Then, 30 *μ*g of the total proteins was isolated by SDS-PAGE and subsequently blotted to the PVDF membrane. The membrane was blocked with a sealing solution containing 5% skimmed milk for 2 h at room temperature. The PVDF membrane was then incubated overnight with a primary antibody of PI3K (dilution of 1 : 500), Akt (dilution of 1 : 500), Bcl-2 (dilution of 1 : 300), Bax (dilution of 1 : 500), cleaved- (C-) caspase-9 (dilution of 1 : 500), C-caspase-3 (dilution of 1 : 500), p-PI3K (dilution of 1 : 500), and p-Akt (dilution of 1 : 500) at 4°C. Subsequently, the PVDF membrane was incubated at room temperature for 1 h using a HPR combined secondary antibody (dilution of 1 : 1000). Finally, the enhanced chemiluminescence (ECL) was used to display the protein bands and record the images. ImageJ software (version 1.51, National Institutes of Health, MD, USA) was used to calculate the gray value of the protein bands collected, and the normalized analysis was carried out.

### 2.18. Statistical Analysis

The chi-squared test was used to analyze the significance of anticonvulsant effect against PTZ- and strychnine-induced seizures among groups (*seizure* rate (%) and death rate (%)). All other data were presented as mean ± standard deviation, and statistical analyses were performed using the two-tailed Student's *t* test with a significance level of *p* < 0.05.

## 3. Results

### 3.1. Chemical Constituents of CPM Water Extract

**Figure 3:**
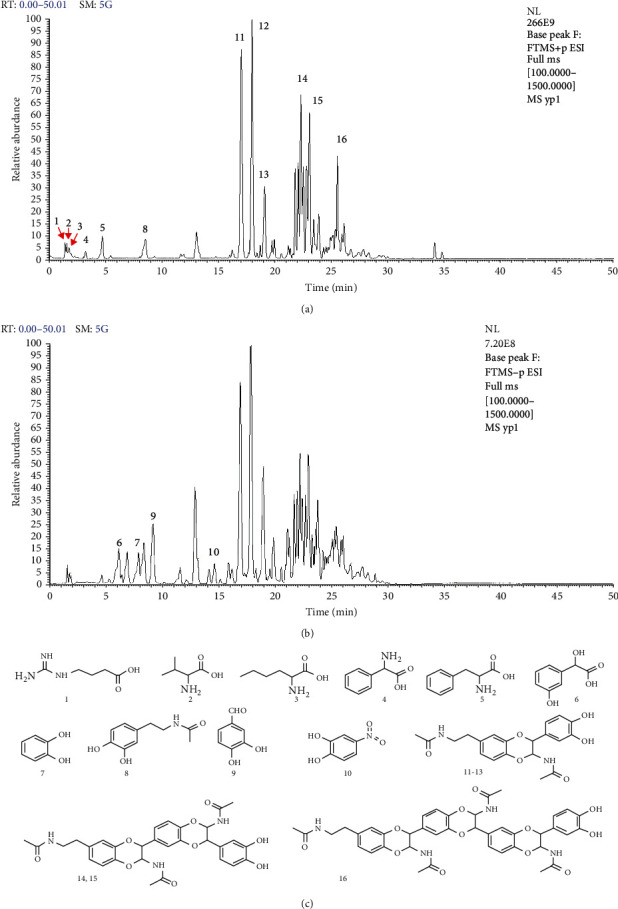
Result of the HPLC-Q Exactive Orbitrap-MS assays of the water extracts of Cicadae Periostracum: (a) MS-FTMS spectrogram (positive mode); (b) MS-FTMS spectrogram (negative mode); (c) compounds in water extracts of Cicadae Periostracum identified by the HPLC-QqQ-MS assays.

Results of the HPLC-Q Exactive Orbitrap-MS assay are shown in Table 1 and Figure 3. In our present study, over 20 peaks were detected by the HPLC-Q Exactive Orbitrap-MS assay within 30 min. As shown in the spectrogram, sixteen major compounds were identified based on composition mass spectrometric behavior and ion fragmentation characteristics and published literature data [17]. These 16 compounds were identified as 4-guanidinobutyric acid (1), L-valine (2), DL-norleucine (3), 2-phenylglycine (4), L-phenylalanine (5), 3-hydroxymandelic acid (6), catechol (7), N-acetyldopamine (8), protocatechualdehyde (9), 4-nitrocatechol (10), and N-acetyldopamine dimers and its isomers (11-13).

### 3.2. CPM Suppressed PTZ- and Strychnine-Induced Seizures in Mice

Effects of CPM on PTZ- and strychnine-induced convulsions in mice are shown in Tables 2 and 3. The present results showed that CPM water extracts at the doses of 200 and 400 mg/kg could prolong the latency of seizure and death of PTZ- and strychnine-induced mice (*p* < 0.05, vs. model mice). In addition, CPM treatments (200 and 400 mg/kg) could also reduce the death rate of PTZ- and strychnine-induced mice (*p* < 0.05 and *p* < 0.01 for the 200 and 400 mg/kg, respectively, vs. model mice). Besides, our results also showed that CPM at 400 mg/kg could decrease the seizure rate of strychnine-induced mice (*p* < 0.05 vs. model mice). These results mentioned above suggested that CPM has the protective effects against chemical drug- (PTZ- and strychnine-) induced seizure in mice.

### 3.3. CPM Possessed Protective Effects against Seizure-Induced Neuron Cell Injury

**Figure 4:**
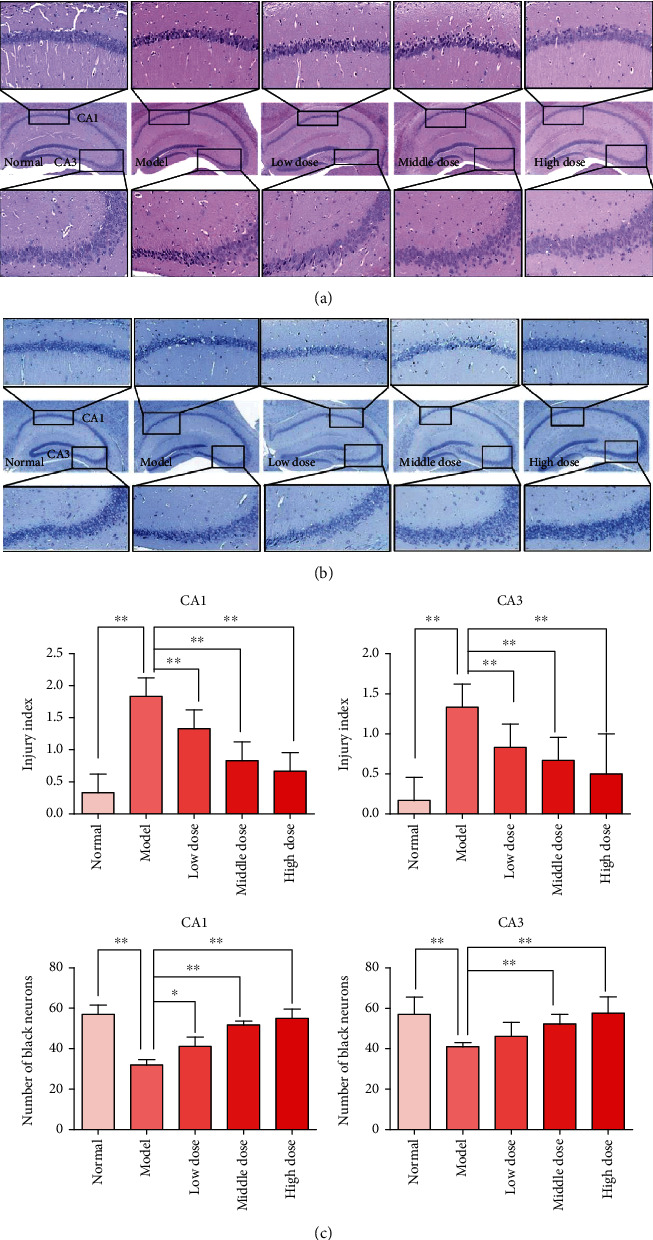
Histological analysis and Nissl staining analysis of hippocampal CA1 and CA3 areas (100x and 400x): (a) H&E staining; (b) Nissl staining; (c) statistical charts for injury index and number of black neurons. Mice were divided into five groups: normal group (normal mice were treated with normal saline alone, 20 mL/kg, p.o.), model group (mice were treated with PTZ and normal saline), and three tested CPM groups (mice were treated with PTZ and CPM at the doses of 100, 200, and 400 mg/kg, p.o.). Data were expressed as mean ± SD (*n* = 5), ^∗^*p* < 0.05 and ^∗∗^*p* < 0.01 vs. model.

As shown in Figure 4, results of the histological analysis were presented. From our results, we can see that no obvious pathological changes can be observed in the hippocampus of normal mice (including cell morphology, cell number, and distribution of hippocampal cells). However, obvious pathological changes could be found in brain tissues of mice with PTZ induction, including irregular cell morphology, disorganized cell arrangement, decreased cell number, and cell nuclear pyknosis in the areas of CA1 and CA3. Interestingly, our results suggested that after pretreatment with CPM at the doses of 200 and 400 mg/kg, the pathological changes of the seizure mice in the hippocampus could be significantly improved with the reduced damage indexes compared to the model mice (*p* < 0.01 vs. model mice).

In addition, we also determined the cell number of dark neurons in the hippocampus with the toluidine blue stain (Figure 4). The results showed that the number of dark neurons in the hippocampus of seizure mice decreased obviously (*p* < 0.01 vs. normal mice), and the CPM at doses of 200 and 400 mg/kg could increase the number of dark neurons in the hippocampus of seizure mice (*p* < 0.05 and *p* < 0.01, respectively), compared to the model mice. All these results mentioned above suggested that CPM possesses protective effects against seizure-induced neuron cell injury in the hippocampus of mice.

### 3.4. CPM Regulated the Expression of Apoptosis-Related Proteins in Mouse Hippocampus

**Figure 5:**
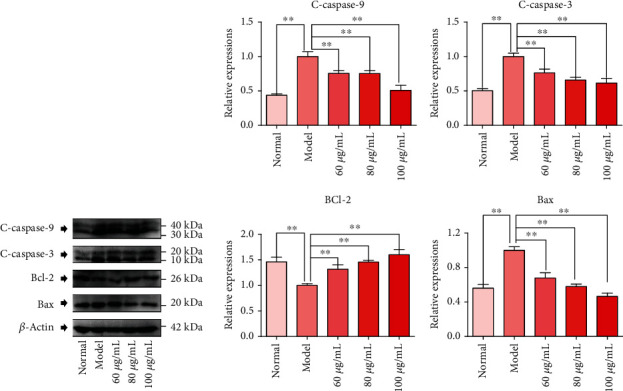
Effect of CPM on the expression level of apoptosis-related proteins in the hippocampus of mice induced by PTZ. Rats were divided into five groups: normal group (normal mice were treated with normal saline alone, 20 mL/kg, p.o.), model group (mice were treated with PTZ and normal saline), and three tested CPM groups (mice were treated with PTZ and CPM at the doses of 100, 200, and 400 mg/kg, p.o.). Hippocampus tissues were collected, and the expression of all proteins was detected by western blotting. Data were expressed as mean ± SD(*n* = 3), ^∗^*p* < 0.05 and ^∗∗^*p* < 0.01 vs. model.

As shown in Figure 5, the expression levels of proapoptotic proteins, including C-caspase 3, C-caspase 9, and Bax, in the hippocampus of normal mice were low, while these proteins were significantly upregulated in PTZ-induced epilepsy mice. Interestingly, the expression of C-caspase 3, C-caspase-9, and Bax decreased after CPM treatment. In addition, CPM also significantly increased the expression of the antiapoptotic protein Bcl-2 in the hippocampus of epileptic mice.

### 3.5. CPM Possessed Cytoprotective Effects on H_2_O_2_-Treated PC12 Cells

**Figure 6:**
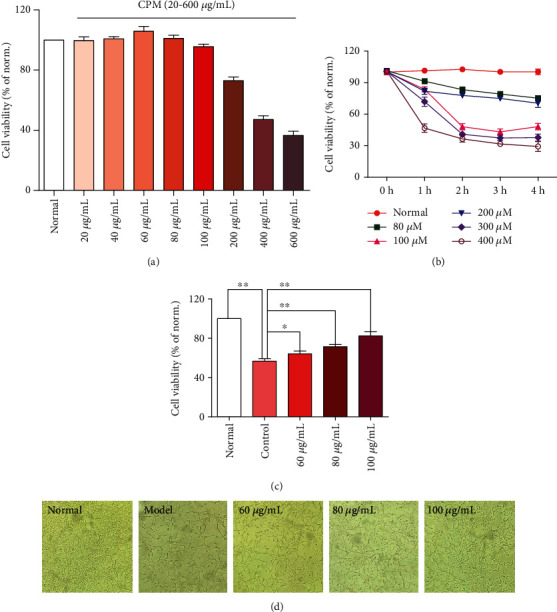
Protective effects of CPM on H_2_O_2_-induced PC12 cells. (a) Effects of CPM on cell viability of normal PC12 cells. (b) Effects of different concentrations and treatment times of H_2_O_2_ on cell viability of PC12 cells. (c) Effects of CPM on cell viability of H_2_O_2_-induced PC12 cells. (d) The represented cell morphology of H_2_O_2_-induced PC12 cells with CPM (×100). Data were expressed as mean ± SD (*n* = 5), ^∗^*p* < 0.05 and ^∗∗^*p* < 0.01 vs. model.

As shown in Figure 6(a), no obvious cytotoxicity on PC12 cells was observed when the concentration of CPM was less than 100 *μ*g/mL. However, over 100 *μ*g/mL, the viability of PC12 cells was greatly affected. Therefore, we selected the CPM at concentrations of 60 *μ*g/mL, 80 *μ*g/mL, and 100 *μ*g/mL as the working concentrations for further investigation of the possible molecular mechanisms of CPM.  Figure 6(b) showed the effects of different concentrations of H_2_O_2_ on the viability of PC12 cells at different times. According to the experimental results, we choose 200 *μ*M as the best working concentration of H_2_O_2_, and the best working time is 4 h (IC_50_ = 198.81). Compared with the normal group, the activity of PC12 cells decreased by about 40% after 4 h stimulation with 200 *μ*M H_2_O_2._ As shown in Figures 6(c) and 6(d), in subsequent experiments, we found that CPM could increase the cell viability of H_2_O_2_-treated PC12 cells, compared with the model group in a dose-dependent manner. These results indicated that CPM inhibited H_2_O_2_-treated PC12 cell injury.

### 3.6. CPM Suppressed Apoptosis in H_2_O_2_-Treated PC12 Cells

**Figure 7:**
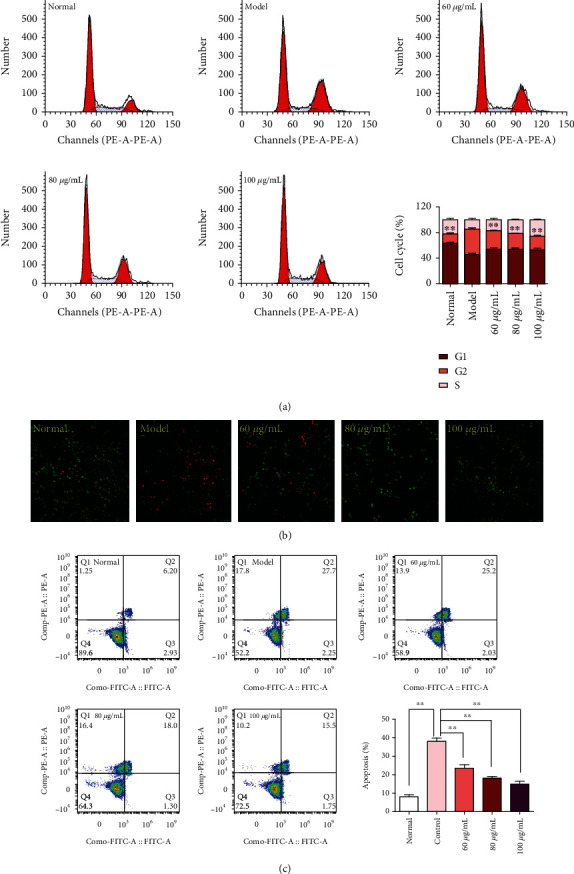
Antiapoptotic effects of CPM on H_2_O_2_-induced PC12 cells. (a) Effects of CPM on cell cycle of H_2_O_2_-induced PC12 cells. (b) Apoptotic assay by AO-EB staining (×100). (c) Apoptotic assay by flow cytometry of PC12 cells. Data were expressed as mean ± SD (*n* = 5), ^∗^*p* < 0.05 and ^∗∗^*p* < 0.01 vs. model.

As shown in Figure 7(a), results of the cell cycle analysis were represented. Our results showed that after stimulation with H_2_O_2_, obvious G2 phase cell arrest was observed, compared to the normal PC12 cells. However, treatment with CPM (60, 80, and 100 *μ*M) can reduce the G2 phase cell arrest in H_2_O_2_-treated PC12 cells (*p* < 0.01, *p* < 0.01, and *p* < 0.01, respectively, vs. the model group). It is reported that G2 phase cell arrest could induce cell apoptosis [18]; therefore, we further determined the effects of CPM on cell apoptosis of H_2_O_2_-treated PC12 cells. To further confirm whether CPM can induce apoptosis or not, AO-EB double staining and FITC conjugated Annexin V/PI staining were carried out by laser confocal microscope and flow cytometry analysis, which are two comprehensively recognized ways for cell apoptosis detection. Interestingly, as we expected, our results suggested that CPM could obviously suppress the H_2_O_2_-treated apoptosis in PC12 cells.

**Figure 8:**
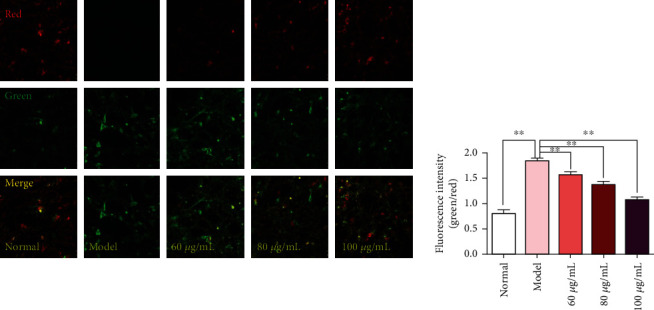
Effects of CPM on the ΔΨm in PC12 cells (×100). Cells were pretreated with CPM (60, 80, and 100 *μ*g/mL) for 2 h and then incubated in the presence of H_2_O_2_ (200 *μ*M) for 4 h. ΔΨm was measured using a JC-1 assay kit and observed using a laser confocal microscopy under a 100x microscope. The red fluorescence indicates that the ΔΨm of the cell is in the normal state, and the JC-1 probe aggregates in the mitochondrial matrix to form a polymer. The green fluorescence indicates that the ΔΨm of the cell decreased, and the JC-1 probe exists in the form of a monomer.

Mitochondrial membrane potential (MOMP, ΔΨm) decline is considered to be an early characteristics of apoptosis cells [16, 19]. In addition, MOMP is of great significance in maintaining the normal physiological function of mitochondria, and the decrease of MOMP is often used as an important indicator of mitochondrial dysfunction. The JC-1 probe is an ideal fluorescent probe widely used to detect the changes of MOMP in cells. Under normal physiological conditions, JC-1 aggregates in the matrix of the mitochondria of cells, forming a polymer that emits red fluorescence. When MOMP is reduced, JC-1 cannot aggregate to the matrix of mitochondria and exists in the form of a monomer, emitting green fluorescence. The changes in MOMP can be detected by comparing the fluorescence intensity changes. Results of the ΔΨm determination in PC12 cells are represented in Figure 8; after being incubated with H_2_O_2_ for 4 h, the red fluorescence in PC12 cells significantly decreased, and the corresponding green fluorescence increased significantly, indicating that the cells had MOMP breakdown. However, compared with the model group, the reduction of red fluorescence in CPM-pretreated cells was less, suggesting that CPM could resist H_2_O_2_-induced MMP loss.

### 3.7. CPM Reduced ROS Level in H_2_O_2_-Treated PC12 Cells

**Figure 9:**
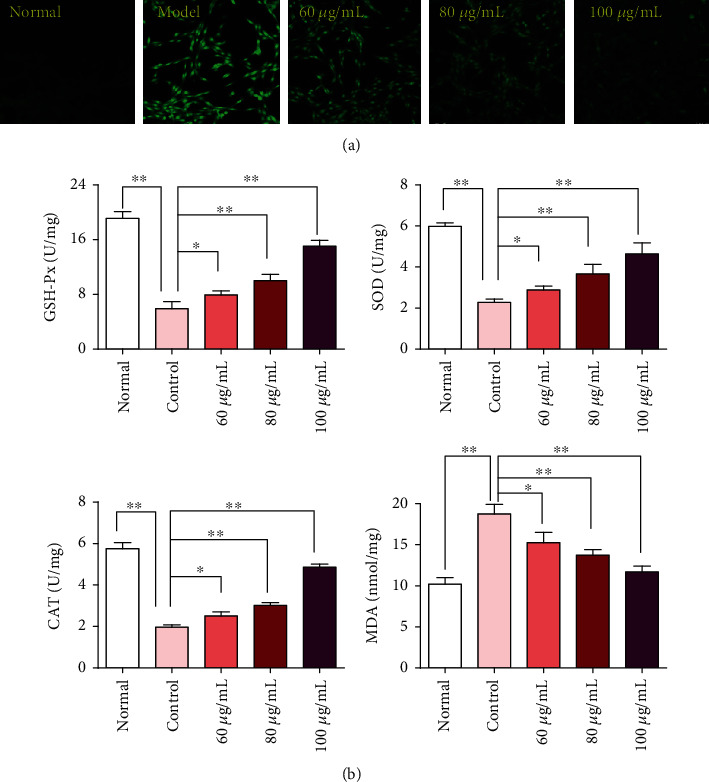
Effects of CPM on ROS levels (a) and antioxidant enzyme activities (b) in H_2_O_2_-induced PC12 cells. PC12 cells were treated with CPM (60, 80, and 100 *μ*g/mL) for 2 h, subsequently subjected to H_2_O_2_ (200 *μ*M) for 4 h. The intracellular ROS levels were observed by laser confocal microscopy. The levels of MDA and activities of SOD, CAT, and GSH-Px were determined by commercial assay kits. Data were expressed as mean ± SD (*n* = 3), ^∗^*p* < 0.05 and ^∗∗^*p* < 0.01 vs. model.

The excessive produced ROS is the primary cause of oxidative damage in an organism [20] We used the DCFH-DA probe to determine the effect of CPM treatment on ROS levels in H_2_O_2_-stimulated PC12 cells. As shown in Figure 9(a), H_2_O_2_ stimulation significantly increased ROS levels in PC12 cells compared with the normal group. Importantly, CPM pretreatment could inhibit ROS levels in H_2_O_2_-treated PC12 cells.

### 3.8. CPM Ameliorated H_2_O_2_-Induced Oxidative Stress in PC12 Cells

When oxidative stress occurs in cells, the intracellular antioxidant enzyme system will be activated to inhibit the excessive production of ROS. SOD, CAT, and GSH-Px are the most important scavenging enzymes of reactive oxygen species in cells. In addition, with regard to lipid peroxidation caused by oxidative stress, the end product of oxidation is MDA, which is cytotoxic. Detection of MDA levels can also assess levels of intracellular oxidative stress [19]. Therefore, in order to evaluate the effect of CPM on H_2_O_2_-induced oxidative stress, we also detected the generation of MDA and the activities of SOD, GSH-Px, and CAT in PC12 cells after intervention. The results are shown in Figure 9(b). Compared with the normal group, the intracellular MDA level in PC12 cells was significantly increased after 4 h H_2_O_2_ stimulation, and the pretreatment with CPM could inhibit MDA production in a concentration-dependent manner. In addition, H_2_O_2_ stimulation can reduce the activities of antioxidant enzymes CAT, GSH, and SOD in cells. Fortunately, CPM pretreatment can enhance the activities of these enzymes. These results suggest that CPM can protect PC12 cells from oxidative damage caused by H_2_O_2_ by increasing the activity of reactive oxygen scavenging enzymes.

### 3.9. CPM Downregulated Apoptotic Proteins Whereas Upregulated Antiapoptotic Proteins

**Figure 10:**
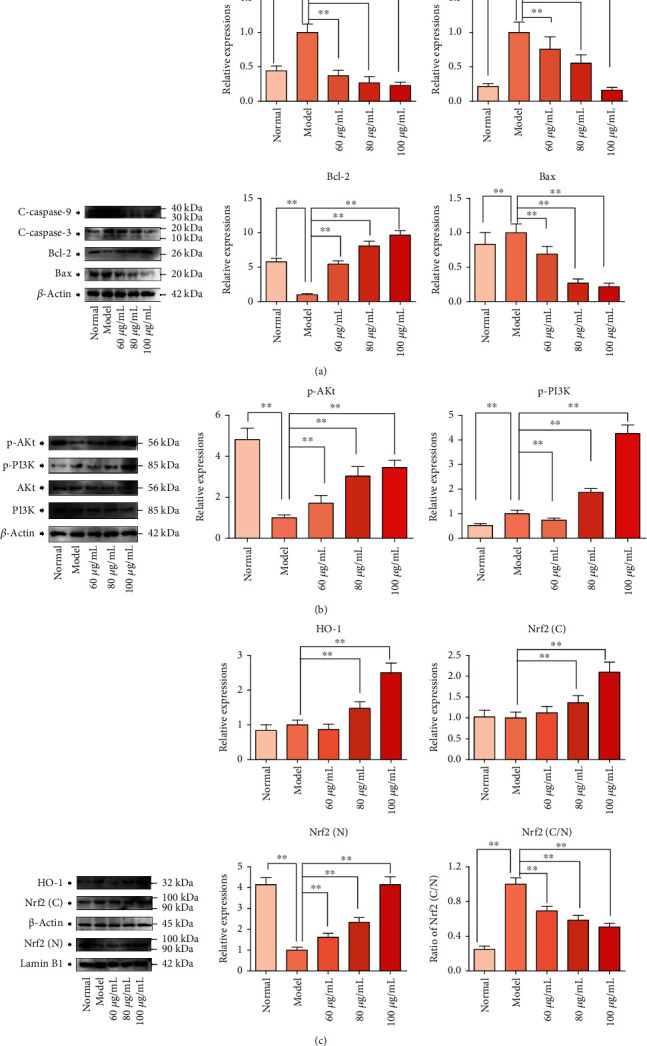
Effects of CPM on PI3K/Akt-Nrf2/HO-1 signaling pathway in H_2_O_2_-induced PC12 cells. (a) Effects of CPM on Bcl-2 and caspase family proteins; (b) effects of CPM on PI3K/Akt signaling; (c) effects of CPM on Nrf2/HO-1 signaling. PC12 cells were treated with CPM (60, 80, and 100 *μ*g/mL) for 2 h, subsequently subjected to H_2_O_2_ (200 *μ*M) for 4 h. All these protein expressions were determined by western blotting assays. Data were expressed as mean ± SD (*n* = 3), ^∗^*p* < 0.05 and ^∗∗^*p* < 0.01 vs. model.

As shown in Figure 10(a), the expressions of Bax, Bcl-2, C-caspase 9, and C-caspase 3 in H_2_O_2_-stimulated PC12 cells were detected by western blot assays. Compared with the normal group, expressions of proapoptotic proteins including Bax (*p* < 0.05), C-caspase 9 (*p* < 0.05), and C-caspase 3 (*p* < 0.05) in H_2_O_2_-treated PC12 cells were obviously upregulated, whereas the antiapoptotic protein of Bcl-2 (*p* < 0.05) was significantly downregulated. However, pretreatment with CPM (60, 80, and 100 *μ*g/mL) increased the Bcl-2 (*p* < 0.05), whereas the expressions of Bax, C-caspase 9 (*p* < 0.05), and C-caspase 3 (*p* < 0.05) in H_2_O_2_-treated PC12 cells were decreased, compared to those of the model PC12 cells.

### 3.10. CPM Activated the PI3K/Akt Pathway in H_2_O_2_-Treated PC12 Cells

The PI3K/Akt pathway is a classical signaling pathway that plays an important role in cell proliferation, differentiation, apoptosis, and other physiological activities. However, only phosphorylation of PI3K and Akt can participate in the regulation of these physiological activities. In addition, increasing studies have found that H_2_O_2_-treated nerve cell apoptosis is related to the inhibition of the PI3K/Akt pathway. Therefore, we evaluated the effect of CPM on the PI3K/Akt pathway in PC12 cells. The experimental results are shown in Figure 10(b). H_2_O_2_ stimulation could significantly inhibit the phosphorylation of PI3K and Akt in PC12 cells, but CPM pretreatment could weaken the inhibitory effect of H_2_O_2_ on Akt phosphorylation. Although low doses of CPM did not appear to activate PI3K, 80 *μ*g/mL and 100 *μ*g/mL of CPM significantly increased PI3K phosphorylation. In addition, CPM and H_2_O_2_ treatment appeared to have little effect on the expression of prototype PI3K and Akt compared to the normal group. These results suggest that CPM can activate the PI3K/Akt pathway in H_2_O_2_-treated PC12 cells.

### 3.11. CPM Activated the Nrf2/HO-1 Pathway in H_2_O_2_-Treated PC12 Cells

**Figure 11:**
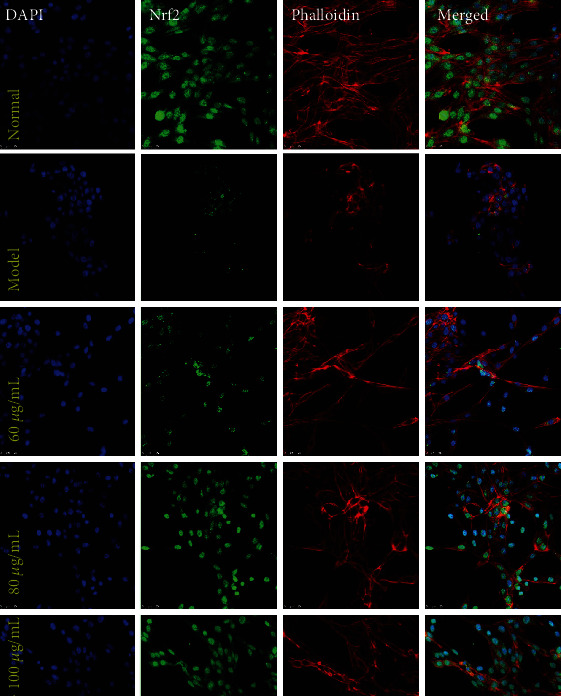
Effects of CPM on nuclear transcription of Nrf2 in H_2_O_2_-induced PC12 cells. PC12 cells were treated with CPM (60, 80, and 100 *μ*g/mL) for 2 h, subsequently subjected to H_2_O_2_ (200 *μ*M) for 4 h. The nuclear transcription of Nrf2 was determined by laser confocal microscopy.

The Nrf2/HO-1 signaling pathway is one of the major antioxidant pathways in cells, which can regulate the intracellular ROS levels [21]. Thus, we speculated that protective effects of CPM against H_2_O_2_-treated oxidative damage in PC12 cells were related to regulation of the Nrf2/HO-1 signaling pathway. In our present study, immunofluorescence and western blot assays were carried out to determine the effect of CPM on the Nrf2/HO-1 signaling pathway in PC12 cells. As we expected, the results showed that after H_2_O_2_ stimulation, Nrf2 nuclear translocation was reduced compared to normal PC12 cells (Figure 11). Interestingly, our results also revealed that CPM pretreatment can increase the Nrf2 nuclear translocation in H_2_O_2_-stimulated PC12 cells (Figure 11). In addition, by fluorescence staining of the cytoskeleton, we found that the cytoskeleton of PC12 cells was significantly damaged after H_2_O_2_ stimulation; however, this damage could be alleviated by CPM pretreatment (Figure 11).

Furthermore, we also determined the protein expressions of Nrf2 and HO-1 in PC12 cells (Figure 10(c)). Our results showed that the expression of Nrf2 in the nucleus of H_2_O_2_-stimulated PC12 cells was significantly decreased (*p* < 0.05), while Nrf2 in the cytoplasm was not significantly affected. Interestingly, CPM pretreatment upregulated nuclear translocation of Nrf2 (*p* < 0.05) in a dose-dependent manner, and CPM could also significantly upregulate (*p* < 0.05) the HO-1 expressions in the cytoplasm (except in the low-dose group) of H_2_O_2_-stimulated PC12 cells, compared to the model PC12 cells.

## 4. Discussion

Traditional Chinese medicines (TCMs) have been used in China to treat various diseases for thousands of years, and accumulating evidences have confirmed that TCMs are beneficial for the control and treatment of some intractable and chronic diseases such as mental disorders [22, 23]. In addition, TCMs are suitable for the diseases which need long-term drug administration due to their relatively less toxicity compared to synthetic drugs [24]. To the best of our knowledge, our present work is the first systemic investigation regarding the antiepileptic effects of CPM and the potential molecular mechanisms.

Based on the experimental epileptic animal models, we have investigated the antiepileptic effects of CPM on PTZ- and strychnine-induced seizures in mice. Interestingly, our present results showed that CPM can prolong the seizure latency and death latency of mice induced by PTZ and strychnine, in a dose-dependent manner. Furthermore, the results also revealed that CPM could reduce the seizure rate and death rate of mice induced by PTZ and strychnine. It is considered that the hippocampus is a vulnerable area during the epileptic seizure due to excitotoxicity, in particular the CA1 and CA3 regions [11, 25]. Furthermore, we have further observed the effects of CPM on pathological changes of the hippocampus of epileptic animals, and our present results showed that CPM could significantly ameliorate the pathological changes of the hippocampus in epileptic mice, including alleviating injury score and protecting the dark neurons.

**Figure 12:**
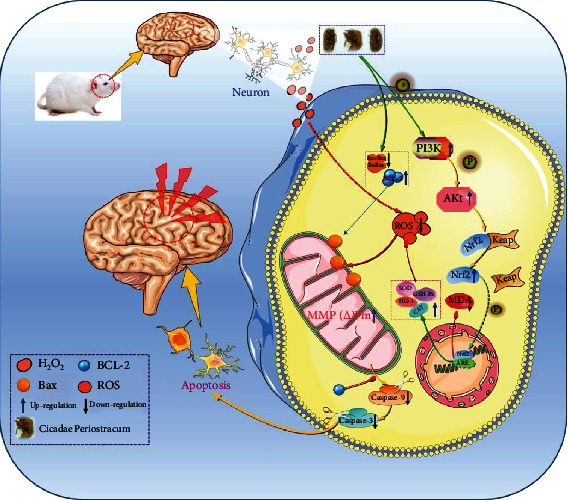
The potential molecular mechanisms for CPM. Cicadae Periostracum possesses cytoprotective effects on H_2_O_2_-treated PC12 cells via regulation of PI3K/Akt/Nrf2 signaling pathways.

Results of the animal experiment suggested that the CPM possessed antiepileptic potentials in experimental epileptic animals; however, the detailed molecular mechanisms remain unclear. Consequently, we further explored the possible mechanisms corresponding to the antiepileptic effects of CPM. The PC12 cell line, a rat photochromogenic cell line, which possesses some neuronal properties and similar physiology and pathology of the neuron cells, is a commonly used cell model for investigating the pathological and physiological process of neuron cells [11, 19]. The H_2_O_2_-induced oxidative-damaged PC12 cell is the most commonly used cell model for investigating the molecular mechanisms of some candidate drugs with protective effects on neuron cells. Interestingly, in our present study, it is uncovered that CPM possessed protective effects on PC12 cells treated by H_2_O_2_. In addition, our results showed that CPM treatment can significantly decrease the apoptosis induced by H_2_O_2_ and downregulate the proapoptotic proteins (caspase-3, caspase-9, and Bax), as well as upregulate the antiapoptotic protein of Bcl-2. Apoptosis is a common programmed death for cells, and it is reported that oxidative stress is a predominant way for targeting apoptosis in cells [25]. Reactive oxygen species (ROS), produced in oxidative stress, is an important inducer of apoptosis. The excessive produced ROS can destruct the mitochondrial membrane and result in the decline of ΔΨm and further lead to the external flow of Cytochrome c (Cyt-C) into cytoplasm from the mitochondrion. Then, the caspase-9 protein would be activated by the released Cyt-C, and subsequently, the activated Caspase-9 would further activate the caspase-3, finally triggering the apoptosis event [26–28]. Therefore, we further determined the ROS levels in H_2_O_2_-stimulated PC12 cells with or without CPM intervention. As we expected, after H_2_O_2_ stimulation, the ROS levels in PC12 cells was sharply increased, and interestingly, the CPM treatment significantly reduced the ROS levels in H_2_O_2_-stimulated PC12 cells. Therefore, we want to know the reasons for the scavenging effects of CPM on ROS in H_2_O_2_-stimulated PC12 cells. In living organisms, there are powerful ROS scavenging enzyme systems, and the predominant antioxidative enzymes are the SOD, CAT, GSH-Px, and HO-1. SOD can catalyze the superoxide anion radical into H_2_O_2_, GSH-Px has the ability of changing the toxic peroxides into nontoxic hydroxyl compounds, and CAT can change the H_2_O_2_ into oxygen and water; all these enzymes can maintain the redox balance in living organisms [19, 29]. Furthermore, HO-1 is an important phase II detoxifying enzyme which plays the crucial roles in protecting the organism from damage by oxidative stress. Our present results revealed that CPM treatment can remarkably increase the activities of antioxidative enzymes (SOD, CAT, GSH-Px, and HO-1) in H_2_O_2_-stimulated PC12 cells. Increasing reports have suggested that the PI3K/Akt/Nrf2 signaling pathway plays the predominant role for redox homeostasis in the organism by preventing the production of ROS [30, 31]. It is reported that Nrf2 is an essential protein for the production of antioxidative enzymes. Under normal conditions, the Nrf2 is located in the cytoplasm by Keap 1. Under oxidative stress, the Nrf2 could translocate into the nuclear by dissociating from Keap 1 and bind to the ARE to activate transcription to generate various antioxidative enzymes in the cytoplasm, including SOD, HO-1, CAT, and GSH. The PI3K/Akt signal pathway plays a key role in cell survival and development [32–35]. In addition, in the upstream of Nrf2 signaling, PI3K/Akt is the predominant signaling for activation of Nrf2. In our present results, we observed that CPM treatment could upregulate the Nrf2 and phosphorylation of PI3K and Akt, suggesting that the scavenging effects of CPM on ROS are closely related to regulation of the PI3K/Akt/Nrf2 signaling pathway in H_2_O_2_-stimulated PC12 cells (Figure 12).

In the year of 2008, a study on the anticonvulsant effect of CPM, the author found that extracts of CPM by ethanol and water have anticonvulsant activities in PTZ-treated mice, and anticonvulsant effects of the water extracts is better than that of ethanol extracts [10]. So, in our present study, we have also investigated the possible active substances in water extracts of CPM and found that the main components in CPM are acetyldopamine polymers and phenols, which is consistent with previous literatures [17]. Previous researches have revealed that acetyldopamine and its polymers in CPM possess promising antioxidant, antidiabetic, and anti-inflammatory activities and regulatory activities on Th1 and Th17 cell differentiation [8, 9, 36, 37]. However, in an earlier study in 1991, it is reported that CPM also contains lots of microelements such as calcium (Ca), magnesium (Mg), and phosphorus (P), and the author thought these microelements might be also related to the anticonvulsant activities of CPM [38]. Consequently, more systemic works should be devoted to investigation of the possible substance basis of CPM for its antiepileptic effect, in particular, the acetyldopamine polymers in CPM.

## 5. Conclusion

In conclusion, our present study suggested that Cicadae Periostracum (CPM) possesses antiepileptic effects through antiapoptosis of neuron cells via regulation of the PI3K/Akt/Nrf2 signaling pathway.

## Figures and Tables

**Table 1:** Precursor and product ions of constituents in Cicadae Periostracum.

No.	Compound name	*t*_R_ (min)	Molecular formula	[M-H]^−^	[M+H]^+^	MS/MS (m/z)
1	4-Guanidinobutyric acid	1.75	C_5_H_11_N_3_O_2_		146.0924	128.0820 [M+H-H_2_O]^+^ 129.0661 [M+H-NH_3_] ^+^
2	L-Valine	1.93	C_5_H_11_NO_2_		118.0866	
3	DL-Norleucine	2.12	C_6_H_13_NO_2_		132.1021	
4	2-Phenylglycine	3.28	C_8_H_9_NO_2_		152.0705	134.0602 [M+H-H_2_O]^+^ 135.0443 [M+H-NH_3_] ^+^
5	L-Phenylalanine	4.77	C_9_H_11_NO_2_		166.0862	
6	3-Hydroxymandelic acid	6.34	C_8_H_8_O_4_	167.0343		335.0773 [2M-H]^−^
7	Catechol	7.04	C_6_H_6_O_2_	109.0286		
8	N-Acetyldopamine	8.53	C_10_H_13_NO_3_		196.0968	391.1862 [2M+H]^+^
9	Protocatechualdehyde	9.34	C_7_H_6_O_3_	137.0237		
10	4-Nitrocatechol	13.30	C_6_H_5_NO_4_	154.0141		
11	N-Acetyldopamine dimer	17.06	C_20_H_22_N_2_O_6_		387.1562	328.1182; 269.0821; 194.0811; 192.0653
12	N-Acetyldopamine dimers	18.00	C_20_H_22_N_2_O_6_		387.1558	328.1178; 269.0813; 194.0808; 192.0649
13	N-Acetyldopamine dimers	19.10	C_20_H_22_N_2_O_6_		387.1556	328.1180; 269.0819; 194.0811; 192.0654
14	N-Acetyldopamine trimer	22.33	C_30_H_31_N_3_O_9_		578.2147	192.0658; 150.0554
15	N-Acetyldopamine trimers	23.06	C_30_H_31_N_3_O_9_		578.2144	192.0661; 150.0559
16	N-Acetyldopamine tetramers	25.54	C_40_H_40_N_4_O_12_		769.2725	192.0664; 150.0563

**Table 2:** Antiepileptic effect of CPM on PTZ induced seizure in mice.

Treatment	Seizure latency (s)	Death latency (s)	Seizure rate (%)	Death rate (%)
Model	83.56 ± 22.51	197.28 ± 30.18	100	100
Positive	1800 ± 0^∗∗^	1800 ± 0^∗∗^	0∗∗	0^∗∗^
100 mg/kg	85.10 ± 19.26	208.54 ± 31.57	100	100
200 mg/kg	124.25 ± 20.67^∗^	383.10 ± 49.38^∗∗^	100	70^∗^
400 mg/kg	209.90 ± 26.29^∗∗^	452.62 ± 65.12^∗∗^	100	40^∗∗^

PTZ: pentylenetetrazole; CPM: Cicadae Periostracum. Diazepam was used as a positive control. Mice were divided into five groups (*n* = 20): model group (mice were treated with normal saline, 20 mL/kg, p.o.), positive group (mice were treated with diazepam, 4 mg/kg, i.p.), and three tested CPM groups (mice were treated with CPM at the doses of 100, 200, and 400 mg/kg, p.o.). The chi-squared test was used to analyze significance of anticonvulsant effects against PTZ-induced seizures among groups (inhibition (%) and mortality (%)). All other data were presented as mean ± SD (*n* = 20), and statistical analyses were performed using the two-tailed Student's *t* test, ^∗^*p* < 0.05 and ^∗∗^*p* < 0.01 vs. model.

**Table 3:** Antiepileptic effect of CPM on strychnine induced seizure in mice.

Treatment	Seizure latency (s)	Death latency (s)	Seizure rate (%)	Death rate (%)
Model	186.32 ± 21.87	275.21 ± 35.66	100	100
Positive	1800 ± 0^∗∗^	1800 ± 0^∗∗^	0^∗∗^	0^∗∗^
100 mg/kg	198.20 ± 43.67	289.62 ± 29.83	100	100
200 mg/kg	324.16 ± 34.76^∗∗^	367.54 ± 31.78^∗^	80	60^∗^
400 mg/kg	426.27 ± 42.73^∗∗^	458.89 ± 34.29^∗∗^	60^∗^	40^∗∗^

CPM: Cicadae Periostracum. Diazepam was used as a positive model. Mice were divided into five groups (*n* = 20): model group (mice were treated with normal saline, 20 mL/kg, p.o.), positive group (mice were treated with diazepam, 4 mg/kg, i.p.), and three tested CPM groups (mice were treated with CPM at the doses of 100, 200, and 400 mg/kg, p.o.). The chi-squared test was used to analyze significance of anticonvulsant effects against strychnine-induced seizures among groups (inhibition (%) and mortality (%)). All other data were presented as mean ± SD (*n* = 20), and statistical analyses were performed using the two-tailed Student's *t* test, ^∗^*p* < 0.05 and ^∗∗^*p* < 0.01 vs. model.

## Data Availability

The data used to support the findings in this paper are available from the corresponding author upon request.
